# Impact of Long-Term Glucocorticoid Therapy on the Response to Biologic Agents in Rheumatoid Arthritis: A Prospective Observational Study

**DOI:** 10.7759/cureus.98824

**Published:** 2025-12-09

**Authors:** Mahamadou Sagara, Abderrahim Majjad, Hamza Toufik, Bezza Ahmed

**Affiliations:** 1 Rheumatology, Mohammed V Military Teaching Hospital, Rabat, MAR

**Keywords:** anti-tnf therapy, biologic dmards, corticosteroids, drug tolerance, glucocorticoid exposure, jak inhibitors, metabolic and bone complications, rheumatoid arthritis, rituximab, treatment efficacy

## Abstract

Background

Prolonged glucocorticoid use remains common in rheumatoid arthritis (RA), particularly in resource-limited settings. However, long-term exposure may reduce the effectiveness of biologic disease-modifying antirheumatic drugs (bDMARDs) and increase the risk of metabolic and skeletal complications. This prospective observational study evaluated the association between cumulative glucocorticoid dose and clinical response to biologic therapies in patients with RA.

Methodology

Adults with RA fulfilling the 2010 ACR/EULAR classification criteria and treated with biologic therapy were prospectively included. Sociodemographic data, disease duration, Disease Activity Score in 28 joints (DAS28), radiographic erosions, inflammatory markers, background and concomitant therapies, and major cardiometabolic and skeletal comorbidities were recorded. Glucocorticoid exposure was analyzed according to treatment duration and cumulative prednisone-equivalent dose. Treatment effectiveness and safety were assessed at 6 and 12 months.

Results

A total of 153 patients were included, predominantly women (75.8%), with a mean age of 57.5 ± 13.7 years and a mean RA duration of 15.2 ± 9.3 years. At baseline, 70.6% were receiving glucocorticoids. Compared with non-exposed patients, glucocorticoid-exposed patients had higher disease activity (mean DAS28-CRP: 4.08 vs 3.09; p = 0.019), a less favorable clinical response at 12 months (DAS28-CRP: 3.92 vs 2.92; p = 0.005), and more radiographic erosions (74.0% vs 35.5%; p = 0.008). The effectiveness of anti-TNF and anti-IL-6 therapies decreased with increasing cumulative glucocorticoid dose, with no good clinical response observed beyond 20,000 mg, whereas response was partially preserved with Janus kinase (JAK) inhibitors (20%) and rituximab (25%). Diabetes (relative risk [RR] = 4.05), hypertension (RR = 4.02), and osteoporosis (RR = 2.48) were more frequent in glucocorticoid-exposed patients.

Conclusions

Prolonged glucocorticoid exposure in RA was associated with a lower likelihood of achieving a good clinical response to biologic therapies targeting cell surface receptors and with higher rates of metabolic and skeletal complications. These real-world data support rational, time-limited glucocorticoid use and careful selection of biologic class in patients with high cumulative exposure.

## Introduction

Rheumatoid arthritis (RA) is a chronic systemic autoimmune disease characterized by persistent synovial inflammation, leading to progressive joint destruction, functional disability, and increased mortality in the absence of early and effective treatment [1-3].

Over the past two decades, the advent of targeted biologic therapies has profoundly transformed the management of RA. Biologic agents such as tumor necrosis factor (TNF) inhibitors, interleukin-6 (IL-6) receptor antagonists (anti-IL-6), anti-CD20 monoclonal antibodies (rituximab), and Janus kinase (JAK) inhibitors have enabled high rates of remission or sustained low disease activity [4-6].

International recommendations from the American College of Rheumatology (ACR) and the European League Against Rheumatism (EULAR) advocate the use of glucocorticoids as short-term adjunctive therapy, in combination with conventional synthetic disease-modifying antirheumatic drugs (csDMARDs), in order to achieve rapid control of inflammation [7-9].

However, in routine clinical practice, especially in resource-limited settings or when access to biologic agents is delayed, glucocorticoid therapy often extends beyond the recommended duration for both clinical and socioeconomic reasons [10].

The adverse effects of chronic glucocorticoid exposure are well documented, particularly metabolic, cardiovascular, and skeletal complications, even at low doses [11,12]. In contrast, their impact on the effectiveness of biologic therapies remains less well defined, especially in cases of prolonged exposure or high cumulative doses.

From a mechanistic standpoint, experimental data suggest that glucocorticoids may attenuate the effectiveness of biologic agents by altering the expression of membrane receptors targeted by these therapies (TNF-R, IL-6R) and disrupting immune signaling pathways [13-15].

In this context, it is essential to better understand the influence of long-term corticosteroid treatment on the efficacy and safety of different classes of biologic agents in RA under real-world clinical conditions. Current pathophysiological hypotheses suggest that some agents, such as JAK inhibitors, which act downstream by blocking the intracellular JAK/STAT pathway, and rituximab, which directly targets B lymphocytes, may maintain more stable efficacy in patients chronically exposed to glucocorticoids [16,17].

In addition, the high frequency of glucocorticoid-induced comorbidities, particularly hypertension, diabetes, and osteoporosis, highlights the importance of generating local data to adapt therapeutic strategies to the epidemiological and economic realities of Morocco.

Study objectives

The primary objective of this study was to evaluate the impact of prolonged exposure to corticosteroids on the clinical effectiveness of different classes of biologic therapies (anti-TNF, anti-IL-6, JAK inhibitors, rituximab). The secondary objectives were to compare the incidence of metabolic and skeletal complications between patients exposed and not exposed to corticosteroids and to analyze the influence of long-term corticosteroid therapy on the safety profile of each class of biologic therapy.

## Materials and methods

Study design and population

This was a prospective observational study conducted between January and December 2024 in the Rheumatology Department of Mohammed V Military Teaching Hospital in Rabat, Morocco. All adult patients (≥18 years) followed for RA fulfilling the 2010 ACR/EULAR classification criteria [18] and treated with biologic therapy were screened for eligibility.

Patients were included if they had received at least six consecutive months of biologic treatment prior to inclusion and had complete clinical and biological follow-up data at 12 months. The main exclusion criteria were the presence of another systemic autoimmune disease, an active chronic infection (notably uncontrolled tuberculosis or hepatitis B or C), and a recent history of active malignancy.

Included patients represented a consecutive series fulfilling these criteria, reflecting recruitment under real-life clinical practice conditions.

Therapeutic protocol and definition of corticosteroid exposure

Background treatments were prescribed in accordance with the 2022 EULAR recommendations and national Moroccan guidelines for the management of RA.

The biologic and targeted synthetic DMARDs (bDMARDs and tsDMARDs) used included the following:

Anti-TNF agents: adalimumab 40 mg subcutaneously every two weeks; etanercept 50 mg subcutaneously once weekly; infliximab 3-5 mg/kg intravenously every eight weeks after induction infusions at weeks 0, 2, and 6.

Anti-IL-6 agents: tocilizumab 8 mg/kg intravenously every four weeks or 162 mg subcutaneously once weekly.

Anti-CD20 agent: rituximab 1 g intravenously on days 1 and 15, repeated every six to 12 months according to clinical and biological response.

JAK inhibitors: tofacitinib 5 mg orally twice daily.

Some patients received successive biologic agents because of primary treatment failure, secondary loss of response, or treatment intolerance. Consequently, the sum of exposure percentages for each biologic class may exceed 100%, as these treatments were not mutually exclusive.

csDMARDs were continued when tolerated: methotrexate 10-25 mg per week (oral or subcutaneous) with folic acid 5 mg weekly; leflunomide 20 mg daily; or sulfasalazine 2-3 g daily.

The choice of biologic class, dosing regimen, and any treatment switches was not standardized but left to the discretion of the treating rheumatologist, based on disease activity, comorbidities, prior therapeutic failures, and reimbursement constraints. This therapeutic approach reflects real-world clinical practice rather than randomized treatment allocation.

Corticosteroid exposure was mainly based on oral prednisone or equivalent, with a usual mean daily dose of ≤10 mg. Prolonged exposure was defined as corticosteroid use for at least six months during the follow-up period. For each patient, the total cumulative dose was calculated in milligrams of prednisone equivalent from doses recorded in the medical chart over the entire treatment period. For analysis, cumulative dose was categorized into three classes: <10,000 mg, 10,000-20,000 mg, and >20,000 mg. Duration of corticosteroid exposure was categorized as <6 months, 6-12 months, and >12 months. Progressive tapering of glucocorticoid therapy was recommended in accordance with EULAR guidelines, ideally within the first six months after initiation, whenever clinically feasible.

Clinical follow-up and assessment of effectiveness

Patients were evaluated at baseline and after 12 months of follow-up. At each visit, a standardized clinical assessment was performed, including a 28-joint tender and swollen joint count, patient and physician global assessments of disease activity using a 0-100 mm visual analog scale (VAS), and measurement of serum C-reactive protein (CRP).

Disease activity was assessed using the Disease Activity Score in 28 joints based on CRP (DAS28-CRP), calculated according to the original methodology described by Prevoo et al. [19]. Use of the DAS28-CRP score complied with the licensing conditions established by MAPI Research Trust [20]; an official authorization request was submitted and is included among the documents provided to the journal.

Additionally, the Clinical Disease Activity Index (CDAI), the Simplified Disease Activity Index (SDAI), and the Health Assessment Questionnaire (HAQ) disability index were calculated according to international recommendations.

A good clinical response at 12 months was defined according to the EULAR response criteria as an improvement in DAS28-CRP of at least 1.2 points from baseline and/or achievement of DAS28-CRP <2.6 (remission).

Response to the different classes of biologic therapy (anti-TNF, anti-IL-6, JAK inhibitors, rituximab) was evaluated according to these criteria, taking into account cumulative corticosteroid exposure.

Safety assessment

Safety assessment was based on the prospective collection of metabolic and skeletal adverse events occurring during follow-up. Metabolic complications included arterial hypertension, diabetes mellitus, and dyslipidemia. Skeletal complications corresponded to osteoporosis confirmed by bone densitometry.

For each patient, cardiometabolic conditions (hypertension, diabetes, dyslipidemia) and osteoporosis were classified as present at baseline (pre-existing) or newly developed during the 12-month follow-up in order to distinguish prior comorbidities from adverse events emerging under treatment. The occurrence of at least one metabolic or skeletal adverse event during follow-up was also coded as a composite variable.

Data on adverse events and comorbidities were collected from medical charts, hospitalization reports, and results of complementary investigations.

The following demographic and clinical variables were also systematically collected: age, sex, body mass index (BMI), disease duration, presence of radiographic erosions, disease activity scores (DAS28-CRP, CDAI, SDAI), HAQ score, immunological status (anti-citrullinated protein antibodies, rheumatoid factor), prior exposure to biologic therapies (first-line biologic vs. successive biologics), concomitant csDMARD use, and biologic class. These variables were incorporated into the analysis to account for potential confounders that could influence both corticosteroid use and the risk of metabolic or skeletal complications.

Ethical considerations

This was a non-interventional prospective observational study conducted in accordance with the Declaration of Helsinki (2013). In line with the institutional policy of Mohammed V Military Teaching Hospital, this type of study is exempt from formal approval by a research ethics committee. A signed statement from the head of the Rheumatology Department confirming the ethical exemption was attached to this revised submission to clarify and document the ethical compliance of the study. Written informed consent was obtained from all participants prior to inclusion.

Statistical analysis

Statistical analyses were performed using JAMOVI software, version 2.26.6 (The Jamovi Project, Sydney, Australia). The distribution of quantitative variables was assessed using the Shapiro-Wilk test. Quantitative variables that did not follow a normal distribution (CRP, corticosteroid dose and duration, DAS28-CRP) were compared using the Mann-Whitney U test. Categorical variables were analyzed using Pearson’s chi-square test or Fisher’s exact test, as appropriate. Results are presented as means ± standard deviation or frequencies (percentages). Analyses were conducted on complete cases without imputation of missing data.

To explore potential treatment selection bias (confounding by indication) between the different biologic classes, baseline demographic, clinical, and therapeutic characteristics (disease activity, presence of erosions, comorbidities, concomitant csDMARD use, prior exposure to biologics) were examined in an exploratory manner across patient groups receiving anti-TNF agents, anti-IL-6 agents, JAK inhibitors, or rituximab. Given the limited size of some subgroups, particularly for JAK inhibitors, and the collinearity between several variables related to disease severity and risk profile, these comparisons were interpreted as descriptive and hypothesis-generating.

For the analysis of clinical response at 12 months, exploratory multivariable models were constructed in the overall cohort, using good clinical response (as defined by EULAR criteria) as the dependent variable. Cumulative corticosteroid exposure and biologic class were the main explanatory variables. Models were adjusted for age, sex, disease duration, baseline DAS28-CRP, prior exposure to biologic therapies (first-line vs. successive biologics), concomitant methotrexate use, and the presence of cardiometabolic comorbidities. These analyses aimed to limit the impact of confounding factors that could influence both corticosteroid use and the likelihood of response to biologic therapies, while taking into account sample size constraints and collinearity between variables. The results of these models were interpreted as exploratory.

For the analysis of metabolic and skeletal adverse events, Poisson regression models with robust variance were used to estimate adjusted relative risks. Corticosteroid exposure (yes/no), exposure duration, and cumulative dose were the main explanatory variables. Models were adjusted for age, sex, BMI, disease duration, baseline DAS28-CRP, presence of pre-existing cardiometabolic comorbidities, and concomitant csDMARD use. Some lifestyle factors, such as smoking status, were not collected in a standardized manner and could not be included in these models; therefore, residual confounding cannot be excluded.

A two-sided p-value <0.05 was considered statistically significant for all analyses.

## Results

Population characteristics

A total of 153 patients with RA were included in the study, most of whom were women (75.8%), with a mean age of 57.5 ± 13.7 years and a mean disease duration of 15.2 ± 9.3 years. The mean BMI was 26.5 ± 4.8 kg/m², reflecting a generally overweight population. Ongoing glucocorticoid therapy was recorded in 70.6% of patients (n = 108).

At baseline, patients exposed to glucocorticoids had significantly higher inflammatory disease activity (mean DAS28-CRP 4.08 vs. 3.09; p = 0.019) and a higher prevalence of radiographic erosions (74.0% vs. 35.5%; p = 0.008) compared with non-exposed patients (Table 1).

**Table 1:** Sociodemographic, clinical, and biological characteristics according to corticosteroid exposure Note: Patients exposed to corticosteroids exhibited significantly higher inflammatory activity and functional disability, as well as an increased frequency of articular erosions. Values in bold indicate statistically significant differences (p < 0.05). BMI, body mass index; DAS28-CRP, Disease Activity Score in 28 joints with C-reactive protein; HAQ: Health Assessment Questionnaire; CRP, C-reactive protein; CDAI, Clinical Disease Activity Index; SDAI, Simplified Disease Activity Index

Variable	Total (n = 153)	Corticosteroid-Exposed (n = 108)	Non-Exposed (n = 45)	p-Value
Age (years), mean ± SD	57.5 ± 13.7	57.8 ± 14.2	57.1 ± 13.0	0.54
Female sex, n (%)	116 (75.8%)	81 (75.0%)	33 (73.3%)	0.76
BMI (kg/m²), mean ± SD	26.5 ± 4.8	26.8 ± 4.7	25.9 ± 4.5	0.55
Disease duration (years), mean ± SD	15.2 ± 9.3	15.3 ± 9.2	15.0 ± 9.3	0.42
DAS28-CRP at baseline	3.79 ± 1.78	4.08 ± 1.80	3.09 ± 1.40	0.019
DAS28-CRP at 12 months	3.75 ± 1.75	3.92 ± 1.80	2.92 ± 1.90	0.005
HAQ	0.96 ± 0.69	1.07 ± 0.90	0.74 ± 0.50	0.009
CRP (mg/L)	19.3 ± 29.3	21.3 ± 11.0	12.1 ± 4.0	0.005
CDAI	15.9 ± 14.6	17.8 ± 13.0	12.0 ± 9.0	0.003
SDAI	17.8 ± 16.4	19.9 ± 15.0	13.6 ± 10.4	0.044
Articular erosions, n (%)	96 (62.7%)	80 (74.0%)	16 (35.5%)	0.008

The most frequent comorbidities were hypertension (29.4%), diabetes mellitus (16.3%), and dyslipidemia (16.3%), all significantly more common among corticosteroid-exposed patients (p < 0.01) (Table 2).

**Table 2:** Comorbidities according to corticosteroid exposure Note: Cardiometabolic comorbidities, particularly hypertension and diabetes mellitus, were significantly more frequent among corticosteroid-exposed patients.

Comorbidity	Corticosteroid-Exposed Patients (n = 108)	Non-Exposed Patients (n = 45)	p-Value
Hypertension	29.4% (32)	8.9% (4)	0.006
Diabetes mellitus	16.3% (18)	4.4% (2)	0.008
Dyslipidemia	16.3% (18)	6.7% (3)	0.012

Regarding background therapy, almost all patients were receiving methotrexate (95.4%), most often in combination with a biologic agent (Table 3).

**Table 3:** Background treatments and biologic therapies according to corticosteroid exposure Note: Methotrexate was the most frequently used conventional DMARD. Anti-TNF agents were significantly more common among corticosteroid-exposed patients, while JAK inhibitors were used exclusively in this group. Values in bold indicate statistically significant differences (p < 0.05). TNF, tumor necrosis factor; IL-6: interleukin-6; JAK, Janus kinase

Treatment	Corticosteroid-Exposed (n = 108)	Non-Exposed (n = 45)	p-Value
Methotrexate, n (%)	105 (97.2%)	41 (91.1%)	0.04
Leflunomide, n (%)	32 (29.6%)	13 (28.9%)	0.94
Sulfasalazine, n (%)	20 (18.5%)	10 (22.2%)	0.62
Anti-TNF, n (%)	85 (78.7%)	25 (55.6%)	0.01
Anti-IL-6, n (%)	28 (25.9%)	7 (15.6%)	0.18
Rituximab, n (%)	69 (63.9%)	23 (51.1%)	0.14
JAK inhibitors, n (%)	5 (4.6%)	0 (0%)	0.03

Therapeutic response and corticosteroid exposure

Patients who received a cumulative glucocorticoid dose <10,000 mg had the highest rates of clinical response, particularly under anti-TNF (70%) and anti-IL-6 (64%) therapies. However, effectiveness decreased progressively with increasing cumulative dose: response rates fell to 29% and 32% for cumulative doses between 10,000 and 20,000 mg and to 0% and 3.5% for anti-TNF and anti-IL-6 agents, respectively, beyond 20,000 mg. JAK inhibitors (20%) and rituximab (24%) retained partial effectiveness despite high cumulative glucocorticoid exposure (Table 4).

**Table 4:** Therapeutic response according to cumulative corticosteroid dose Note: The efficacy of anti-TNF and anti-IL-6 therapies decreased in a dose-dependent manner with increasing cumulative corticosteroid exposure, reaching 0% and 3.5%, respectively, beyond 20,000 mg. Conversely, JAK inhibitors and rituximab maintained partial efficacy even at high corticosteroid doses. TNF, tumor necrosis factor; IL-6, interleukin-6; JAK, Janus kinase

Cumulative Dose (mg prednisone equivalent)	Anti-TNF	Anti-IL-6	JAK Inhibitors	Rituximab
<10,000	70% (60/85)	64% (18/28)	40% (2/5)	50% (35/69)
10,000–20,000	29% (25/85)	32% (9/28)	40% (2/5)	26% (18/69)
>20,000	0% (0/85)	3.5% (1/28)	20% (1/5)	24% (16/69)

A duration of glucocorticoid exposure between 6 and 12 months was associated with a significant reduction in response rates to anti-TNF therapy (relative risk [RR] = 0.60; 95% CI: 0.40-0.91; p = 0.02) and anti-IL-6 therapy (RR = 0.55; 95% CI: 0.33-0.89; p = 0.03). This reduction was even more pronounced beyond 12 months of exposure (RR = 0.45; 95% CI: 0.25-0.80; p = 0.006 for anti-TNF agents; RR = 0.50; 95% CI: 0.27-0.86; p = 0.02 for anti-IL-6 agents) (Table 5).

**Table 5:** Duration of corticosteroid exposure and therapeutic response *p < 0.05; **p < 0.01. Note: A corticosteroid exposure longer than 12 months significantly reduces clinical response to anti-TNF and anti-IL-6 therapies, whereas the efficacy of JAK inhibitors and rituximab remains relatively stable. RR, relative risk; CI, confidence interval; Ref., reference group

Duration of Exposure	Anti-TNF, RR [95% CI]	Anti-IL-6, RR [95% CI]	JAK Inhibitors, RR [95% CI]	Rituximab, RR [95% CI]
<6 months	Ref. (1.00)	Ref. (1.00)	Ref. (1.00)	Ref. (1.00)
6–12 months	0.60 [0.40–0.91]*	0.55 [0.33–0.89]*	0.75 [0.45–1.18]	0.70 [0.40–1.08]
>12 months	0.45 [0.25–0.80]**	0.50 [0.27–0.86]*	0.68 [0.38–1.10]	0.65 [0.36–1.15]

JAK inhibitors and rituximab did not show a significant variation in effectiveness according to the duration of glucocorticoid exposure. Class-wise analysis confirmed a marked loss of effectiveness for anti-TNF and anti-IL-6 agents at cumulative doses >20,000 mg (RR = 0.40 and 0.45, respectively; p < 0.05), whereas JAK inhibitors and rituximab maintained more stable response rates (Table 6).

**Table 6:** Comparison according to the class of biologic therapy Note: The efficacy of anti-TNF and anti-IL-6 agents is significantly more impaired by prolonged corticosteroid exposure compared to JAK inhibitors and rituximab, whose therapeutic response remains better preserved. RR, relative risk; NS, not significant; TNF, tumor necrosis factor; IL-6, interleukin-6; JAK, Janus kinase; CS, corticosteroids

Parameter	Anti-TNF	Anti-IL-6	JAK Inhibitors	Rituximab
Impact of dose > 20,000 mg	RR = 0.40 (p = 0.01)	RR = 0.45 (p = 0.02)	RR = 0.70 (NS)	RR = 0.65 (NS)
Clinical response at 12 months (DAS28-CRP)	3.92 (CS+) vs 2.92 (CS–)	Similar trend	Better preserved	Better preserved
Exposure > 12 months	RR = 0.45 (p < 0.01)	RR = 0.50 (p < 0.05)	RR = 0.68 (NS)	RR = 0.63 (p < 0.05)
Adverse events	↑ metabolic and bone risk (all)	↑ metabolic and bone risk	Similar	Similar
Mechanism of action	TNF blockade	IL-6 receptor blockade	JAK/STAT pathway inhibition	B-cell depletion (CD20)

Adverse events

Prolonged glucocorticoid exposure was associated with an increased frequency of metabolic and skeletal adverse events. Among exposed patients (n = 108), hypertension was observed in 36.1%, osteoporosis in 25.0%, and diabetes in 20.4%. These rates were significantly higher than those observed in non-exposed patients (8.9%, 11.1%, and 6.7%, respectively; p < 0.01).

Adjusted relative risks showed approximately a fourfold increase in the risk of diabetes (RR = 4.05; 95% CI: 1.60-10.25; p = 0.003) and hypertension (RR = 4.02; 95% CI: 1.85-8.70; p = 0.001), and about a 2.5-fold increase in the risk of osteoporosis (RR = 2.48; 95% CI: 1.12-5.48; p = 0.02).

Overall, 39.8% of glucocorticoid-exposed patients experienced at least one metabolic or skeletal complication compared with 15.6% of non-exposed patients (RR = 3.67; 95% CI: 1.55-8.68; p = 0.002), confirming the unfavorable risk profile of prolonged glucocorticoid exposure (Table 7).

**Table 7:** Adverse effects associated with prolonged corticosteroid therapy Note: Patients exposed to corticosteroids had significantly increased risks of diabetes, hypertension, and osteoporosis, confirming the unfavorable safety profile of prolonged corticosteroid therapy in rheumatoid arthritis. RR, relative risk; CI, confidence interval

Adverse Effect	Exposed (n = 108)	Non-Exposed (n = 45)	RR [95% CI]	p-Value
Osteoporosis	27 (25.0%)	5 (11.1%)	2.48 [1.12–5.48]	0.02
Diabetes mellitus	22 (20.4%)	3 (6.7%)	4.05 [1.60–10.25]	0.003
Hypertension	39 (36.1%)	4 (8.9%)	4.02 [1.85–8.70]	0.001
≥1 adverse event	43 (39.8%)	7 (15.6%)	3.67 [1.55–8.68]	0.002

## Discussion

This prospective real-world study conducted in Morocco aimed to evaluate the impact of prolonged glucocorticoid therapy on the effectiveness and safety of biologic agents in patients with RA. In this cohort followed under routine clinical practice conditions, long-term glucocorticoid exposure was associated with a significant reduction in clinical response to anti-TNF and anti-IL-6 therapies, as well as an increased incidence of metabolic and skeletal complications. In contrast, JAK inhibitors and rituximab appeared to retain partial effectiveness despite prolonged glucocorticoid exposure. These findings should be interpreted as real-world associations rather than proof of a causal relationship.

The observed dose-response relationship, with an almost complete loss of response beyond a cumulative prednisone-equivalent dose of 20,000 mg, is consistent with previous reports describing a lower likelihood of achieving remission under biologic treatment in the context of chronic glucocorticoid use, regardless of csDMARD background therapy [16,17]. These data suggest that high cumulative glucocorticoid exposure may represent a marker of lower probability of response to biologic agents targeting TNF or IL-6.

From a mechanistic standpoint, this reduced effectiveness may result from glucocorticoid-induced downregulation of membrane receptors targeted by biologic agents, a phenomenon previously demonstrated in vitro in synovial fibroblasts and activated monocytes [21]. Altered expression or signaling of TNF-R and IL-6R could thus contribute to diminished sensitivity to agents that directly target these receptors.

In contrast, the partial preservation of response under JAK inhibitors and rituximab suggests that their mechanisms of action intracellular pathway blockade for JAK inhibitors (inhibition of the JAK/STAT pathway) and B-cell depletion via CD20 targeting for rituximab may be less sensitive to the long-term immunomodulatory effects of glucocorticoids. These observations are consistent with data showing that baricitinib maintains effectiveness comparable to that of anti-TNF agents even in the presence of glucocorticoids [22] and that responses to rituximab remain broadly stable despite prolonged immunosuppressive treatments [23]. In our cohort, these elements translated into a less pronounced attenuation of clinical response to JAK inhibitors and rituximab among patients with the highest glucocorticoid exposure.

Regarding safety, prolonged glucocorticoid exposure was clearly associated with an increased incidence of metabolic and skeletal adverse events: the risks of diabetes and hypertension were approximately fourfold higher, and the risk of osteoporosis was increased by about 2.5-fold. These findings are in line with numerous studies describing increased cardiovascular and skeletal morbidity associated with long-term glucocorticoid therapy in RA [24,25]. They confirm, in a North African real-world context, the well-established long-term risk profile of glucocorticoids.

Analysis by duration of exposure reinforced these trends: beyond 12 months, the effectiveness of anti-TNF and anti-IL-6 agents was reduced by approximately 50%. These results support the 2022 EULAR recommendations, which emphasize early and progressive tapering of glucocorticoids within the first three to six months of treatment and stress the need to avoid prolonged glucocorticoid therapy whenever possible [26].

Taken together, these findings argue in favor of an individualized therapeutic approach that incorporates each patient’s glucocorticoid exposure profile. In glucocorticoid-dependent patients or those with high cumulative exposure, biologic agents with predominantly intracellular modes of action (JAK inhibitors) or B-cell-depleting agents (rituximab) may offer relatively better-preserved effectiveness, although these observations need to be confirmed in studies including larger numbers of patients treated with these classes. In parallel, implementing structured glucocorticoid tapering strategies and close monitoring of metabolic and skeletal comorbidities appears essential, particularly in resource-limited settings where prolonged glucocorticoid use remains frequent and sometimes difficult to avoid.

Strengths and limitations of the study

This study has several methodological strengths. Its prospective, real-world design in a specialized rheumatology department is appropriate to address the research question and allows description of the associations between prolonged glucocorticoid therapy, response to biologic agents, and complications in routine practice. Inclusion and exclusion criteria based on the 2010 ACR/EULAR classification criteria were clearly defined and applied to a consecutive cohort, supporting transparent recruitment and potential reproducibility. Disease activity and treatment response were assessed using standardized, validated indices (DAS28-CRP, CDAI, SDAI, HAQ) and EULAR response criteria, and data were collected prospectively in a resource-limited setting, which enhances the relevance of the findings for similar health systems.

Several limitations must also be acknowledged. Although the overall sample size is acceptable, numbers become limited in some subgroups, particularly patients treated with JAK inhibitors; thus, analyses by biologic class should be considered exploratory. The monocentric design within a Moroccan tertiary military hospital may restrict generalizability to other countries or levels of care, where patient profiles and prescribing practices may differ. The lack of randomization and the clinician-driven choice, dosing, and switching of biologic agents expose the study to treatment-selection and indication bias; in particular, patients receiving prolonged glucocorticoid therapy had more severe, longstanding, erosive, and disabling RA, which may partly account for lower response rates. Despite multivariable adjustment for major confounders, residual confounding cannot be excluded. Finally, glucocorticoid exposure was reconstructed from medical records and heterogeneous tapering regimens, with possible misclassification, and lifestyle factors such as smoking were not systematically collected. Accordingly, the observed associations between prolonged glucocorticoid exposure, biologic response, and complications should be interpreted as real-world, hypothesis-generating signals rather than proof of causality.

## Conclusions

Prolonged glucocorticoid exposure in patients with RA was associated with a lower likelihood of achieving a good response to anti-TNF and anti-IL-6 therapies, relatively better-preserved effectiveness of JAK inhibitors and rituximab, and an increased rate of metabolic and skeletal complications. Without allowing causal inferences, these real-world data support limiting the duration of glucocorticoid use in patients receiving biologic therapy and suggest that the choice of biologic class may be particularly important in those with high cumulative glucocorticoid exposure. Larger multicenter studies are needed to confirm these observations and to better define the respective roles of the different biologic classes in this context.
